# Crystal structures of low-melting molecular materials

**DOI:** 10.1107/S2052252526011024

**Published:** 2026-11-09

**Authors:** Simon Parsons

**Affiliations:** a EaStCHEM School of Chemistry and Centre for Science at Extreme Conditions University of Edinburgh King's Buildings W. Mains Road EdinburghEH9 3FJ United Kingdom

**Keywords:** propane, propene, neutron diffraction, extraterrestrial compounds

## Abstract

The crystal structures of propane and propene have been studied by Fortes and co-workers [*IUCrJ* (2026), **13**, 689–704] using neutron diffraction, spectroscopy and theoretical calculations. The combination of these methods reveals their crystal structures, thermal expansion, vibrational properties and phase behaviour, with implications for understanding hydro­carbon ices in extraterrestrial environments.

The crystal structures of simple materials form the basis of our understanding of the structures of solids. Very many inorganic materials crystallize in basic structural types such as the NaCl or CaF_2_ structures. The work of Kitaigorodski (Kitaigorodski, 1973 ▸) and Peresypkina & Blatov (Peresypkina & Blatov, 2000*a* ▸; Peresypkina & Blatov, 2000*b* ▸) showed that simple sphere-packing archetypes apply also to molecular materials. The crystal structures of simple molecular solids provide archetypes for intermolecular interactions in more complex materials, while they are also small enough for theoretical modelling at the highest levels.

The Cambridge Structural Database (Groom *et al.*, 2016 ▸) now contains over 1.4 million crystal structures, and yet it is often surprising to discover that structural data on the very simplest compounds have not been determined. Usually this is because they form liquids or gases under normal conditions and therefore special techniques have to be applied for crystallization. For the purposes of single-crystal diffraction experiments, amongst the most successful of these is Boese’s *in situ* crystal growth method in which a focused CO_2_-laser beam is moved along a capillary containing the frozen material of interest while mounted on a diffractometer (Boese & Nussbaumer, 1994 ▸). Crystal growth is achieved through careful control of a liquid–solid phase boundary which is tracked slowly along the length of the sample, somewhat akin to zone refinement. Among Boese’s most highly cited papers is his report of the crystal structures of the primary alkanes, which identified the structural reason for the alternation of melting points along the series (Boese *et al.*, 1999 ▸).

Powder diffraction is an attractive alternative technique for the study of low-melting materials as they usually have quite simple crystal structures. Neutron powder diffraction is particularly attractive, as structures can be determined at temperatures near absolute zero, providing a rigorous benchmark for theoretical work. The HRPD instrument at the ISIS Neutron and Muon Source has a distinguished record in this respect, some personally selected highlights being ozone (Marx & Ibberson, 2001 ▸), stannane (Maley *et al.*, 2008 ▸), pyridine (Crawford *et al.*, 2009 ▸), ammonia hydrates (Fortes *et al.*, 2009 ▸; Griffiths *et al.*, 2012 ▸), iron penta­carbonyl (Fortes & Parker, 2022 ▸) and phosgene (Ringelband *et al.*, 2026 ▸) and its sulfur-containing analogue, thio­phosphene (Tambornino *et al.*, 2024 ▸). This is the technique applied by Fortes, Howard & Parker in their paper on the structures of propane and propene, which appears in this issue of *IUCrJ* (Fortes *et al.*, 2026 ▸).

Propane (C_3_H_8_) and propene (C_3_H_6_) are among the very simplest hydro­carbons. They are found in interstellar environments, planetary atmospheres and icy bodies such as Saturn’s moon, Titan. Understanding their physical properties in the solid state is important for interpreting astronomical infrared spectra and modelling the behaviour of hydro­carbon ice under the extreme conditions of extraterrestrial environments. Prior to this work, the crystal structure of propene had not been reliably determined at all, and even for propane, little was known of its structural response to temperature or its solid-state vibrational properties, especially for the external phonon modes which are critical for modelling thermodynamic properties.

The crystal structures of both compounds have been determined between 10–80 K for propane and 10–85 K for propene (Fig. 1 ▸). The structure determined for propane is the same as that determined by Boese, but, of course, the neutron data provide the H-atom positions with much higher precision and accuracy. The structure of propene forms in *P*2_1_2_1_2_1_ with *Z*′ = 2. Intermolecular energies were estimated for both structures. Whereas propane forms a layered structure, propene forms a three-dimensional arrangement of interlocking, canted molecules. The variable-temperature data enable thermal expansion properties of the solids to be determined for the first time. Expansion is moderately anisotropic for propane, with the greatest effect seen along the direction of the weakest interlayer contact. Expansion of propene is approximately uniaxial, perhaps as a result of the underlying tetragonal symmetry of its crystal structure (see below).

The structural data were obtained for deuterated isotopologues of propane and propene in order to avoid the high backgrounds in the diffraction patterns associated with inelastic scattering from protium. Volume isotope effects arising from replacing hydrogen with deuterium were estimated, enabling the densities of ordinary, hydrogenated propane and propene to be parameterized across their solid-state temperature ranges. These estimates provide useful inputs for planetary models, particularly calculations of whether hydro­carbon ice particles can float in Titan’s liquid hydro­carbon lakes and rivers.

Inelastic neutron scattering (INS) measurements (a form of vibrational spectroscopy), supported by periodic DFT calculations, enabled the assignment of vibrational modes in both hydrogenated and deuterated propane and propene, with excellent agreement between the observed and calculated spectra.

The INS data help resolve the more complex phase behaviour of propane and propene seen using vibrational spectroscopy (Hudson *et al.*, 2021 ▸). Previous investigations have shown that propane exists in two solid forms, a metastable phase I which emerges at 40–50 K from an amorphous solid deposited at 10 K, and a phase II which is formed on warming above 60 K. The observed and calculated INS data clearly identify the structure determined with the spectroscopists’ phase II, and the structure of phase I remains unknown.

Two phases have also been observed spectroscopically for propene, a metastable phase I transforming to a stable phase II above 70 K (Hudson *et al.*, 2021 ▸). A related study along similar lines to the present one was carried out by Lennon *et al.* in 2000 ▸ (Lennon *et al.*, 2000 ▸). No reliable structure solution could be obtained, but the diffraction data were clearly different to those collected in the present study. Reindexing of the old data suggested an alternative tetragonal phase with cell dimensions which are similar but significantly different to the orthorhombic phase determined here. Crystal structure prediction suggests a structure in *I*4 with slightly different relative orientations of the molecules to those in the ortho­rhombic structure. While the INS data collected in this study are consistent with the reported orthorhombic phase, calculated infrared data for the *I*4 phase are more consistent with Hudson’s infrared data for phase I.

The authors point out that these results demonstrate the merits of revisiting old data using modern methods, but it is also a nice example of the detail and insight that can be obtained when diffraction, spectroscopy and theory are combined.

## Figures and Tables

**Figure 1:**
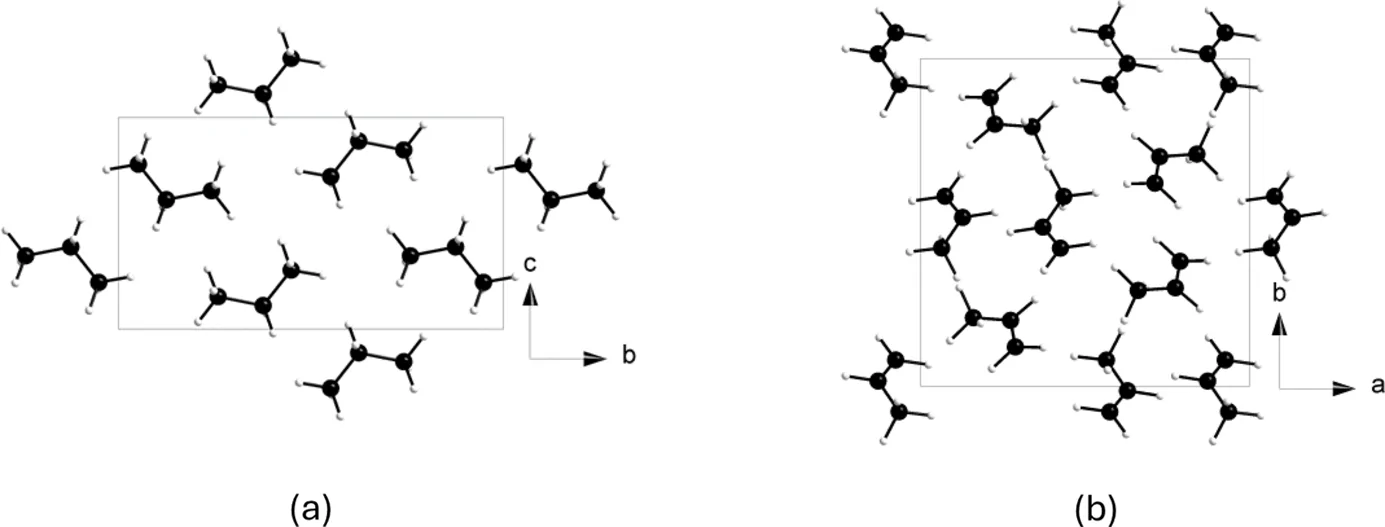
The crystal structures of (a) propane and (b) propene at 10 K.
